# Description of an Equine Hepacivirus Cluster in a Horse Stable in Italy

**DOI:** 10.1155/2023/5251034

**Published:** 2023-05-05

**Authors:** Roberta Cardone, Alessio Buonavoglia, Gianvito Lanave, Violetta Iris Vasinioti, Valentina Mininni, Eleonora Lorusso, Nicola Decaro, Vito Martella, Gabriella Elia, Georgia Diakoudi

**Affiliations:** ^1^Department of Veterinary Medicine, University of Bari Aldo Moro, Valenzano, Italy; ^2^Veterinary Practitioner, Bari, Italy

## Abstract

Equine hepacivirus (EqHV), also known as Hepacivirus A, represents the most closely related genetic homologue of human hepatitis C virus (HCV). Although detected worldwide, limited information on the clinical features of this infection is available and on the mechanisms by which EqHV is transmitted. In this study, we describe a spread of infection of EqHV that occurred in a small stable of horses in southern Italy. The RNA of EqHV was detected in 6/13 (46.2%) sera of the horses introduced into the herd, at different times, over a period of approximately one year. Based on the sequencing analyses of genomic portions located in the NS5B, 5′UTR, and NS3 genes, the viruses detected in the animals were genetically highly related (100% nt identity) to each other. The nearly full-length genome of the virus identified from two horses was generated. For one animal with a profile of chronic infection, the genome sequence was determined with a 7-month interval, revealing 26nt changes resulting in 11 nonsynonymous intrahost nucleotide variations. Overall, based on the epidemiological information, we support the hypothesis that horizontal transmission occurred in the herd.

## 1. Introduction

Human hepatitis C virus (HCV), genus *Hepacivirus*, family *Flaviviridae*, is a hepatotropic virus and a major cause of acute and chronic hepatitis, occurring in up to 3% of world population [1, 2]. For many years, HCV was the only recognized species within the genus. During the last decade, the identification of new hepaciviruses in various mammalian and nonmammalian animal species [3–5], including horses and donkeys [6, 7], has led to new scenarios on the still obscure origin and evolution of the human virus. The discovery of these new viruses required the update of the classification of the genus *Hepacivirus* into 14 species, namely, Hepacivirus A to N [1].

Among the animal hepaciviruses, the equine virus (EqHV) is the closest homolog of HCV sharing both the genome organization and gene functions [8]. EqHV genome is a positive single-stranded RNA of about 9.6 kb in size, with a long unique ORF encoding a polyprotein, precursor of the structural and nonstructural proteins [9]. Similarly to HCV, EqHV displays a predominant liver tropism and is able to establish chronic stage of infection [10–12]. However, unlike HCV, prevalence studies indicate that EqHV persistent infection occurs in <25% of horses [11, 12], and it has not been associated with severe clinical disease such as cirrhosis and hepatocellular carcinoma in humans. This difference in infection outcome makes hepacivirus A, an attractive model system for investigating the determinants of resolving and persistent hepacivirus infections [13].

Epidemiological studies have shown the worldwide spread of EqHV, but the mechanisms of its transmission are still unclear. In horses, transmission of EqHV seemingly resembles that of HCV, i.e., transmission by parenteral route through biological products or infected blood [14] and occasional vertical transmission [15]. However, other routes of transmission are considered likely in order to justify the prevalence data of EqHV worldwide [16–18].

In this study, the identification and the epidemic pattern of EqHV in a small horse stable in South Italy is described.

## 2. Materials and Methods

### 2.1. Serum Samples

Serum and blood samples from a total of 13 trotting horses were collected periodically from a small stable in south Italy, by the stable veterinarian. Starting from 1st March 2021, two 3-year-old castrated horses (#136 and #142) and an 18-month-old foal (#141) exhibited some clinical signs that may be suggestive of liver disease including weight loss, poor sports performance, and altered liver enzyme profile (Supplementary Table 1). The horses had no clinical history of any kind of medical treatment, before and during the sampling period. Periodic sampling of serum, urine, feces, nasal, and oropharyngeal swabs were collected from all the animals in the stable and subsequently from all newly introduced horses for a nearly one-year period. The samples were screened for a panel of horse pathogens (panel HP), that included Equine Infectious Anemia virus [19], Anaplasma spp., Ehrlichia spp., Babesia spp. [20, 21], EqHV, and equine pegiviruses (EPgV and Theiler's disease-associated virus; TDAV) [22]. Biochemistry analyses were performed on sera samples that tested positive to equine viruses associated with hepatic disease.

### 2.2. Viral Nucleid Acid Extraction and RT-PCR for EqHV

Viral RNA and DNA were extracted from all samples using the IndiSpin® Pathogen Kit (Indical Bioscience GmbH, Leipzig, Germany) according to the manufacturer's instructions. Viral nucleid acids extracted were conserved in −80°C till use.

All RNA extracts were tested for EqHV using a quantitative real time reverse transcriptase (RT)-(q) PCR targeting the 5′ UTR genomic region, as previously described [6, 9]. The extracts were also tested for other pathogens of the diagnostic panel HP as described previously [19–22].

Sera samples positive to EqHV in RT-qPCR were further amplified in conventional RT-PCR assays set up on conserved regions of NS3, NS5B, and 5′ UTR [6]. The PCR products obtained were purified, cloned, and sent for direct sequencing to Eurofins Genomics GmbH (Ebersberg, Germany).

### 2.3. MinION Sequencing and Data Analysis

In order to obtain the complete viral sequence of the EqHV strain circulating in the stable, samples were selected from two animals, horse #142 that displayed a profile of persistent infection and horse #359 (strain ITA/2021/359) based on the viral RNA copy number (RNA > 10^4^ copies/10 mL). The EqHV strain from horse #142 was submitted to whole genome sequencing twice, using a sample collected in March 2021 (strain ITA/2021/142 m), at the beginning of the infection, and a sample collected in October 2021 (strain ITA/2021/142o), 7 months later, in order to observe possible within-host viral evolution.

The samples were initially treated with TURBO™ DNase (ThermoFisher Scientific, US) and incubated at 37°C for 30 min and then the RNA was purified using Zymo research RNA Clean & Concentrator™-5 with Zymo-Spin™ IC columns (Zymo Research, UK) according to manufacturer's instructions. Viral RNA was enriched by RT-PCR protocols using overlapping primers specific for EqHV covering the almost complete EqHV genome (Supplementary Table 2). cDNA was generated using SuperScript III First-Strand Synthesis SuperMix (ThermoFisher Scientific, US) and PCRs were performed using TaKaRa LA Taq DNA polymerase (TaKaRa Bio, Japan).

One *μ*g of equimolar pooled PCR products for each EqHV sample was the input to the libraries prepared using Ligation Sequencing Kit (SQK-LSK110, Oxford Nanopore Technologies) according to manufacturer's protocol. The libraries were purified using Agencourt AMPure XP beads (Beckman Coulter, CA, USA) and the final concentration was quantified using a Qubit 2.0 Fluorometer (Thermo Fisher Scientific). The libraries were sequenced independently using R9.4.1 FLO-MIN106D flowcells (Oxford Nanopore Technologies) on MinION Mk1C platform (Oxford Nanopore Technologies) for 12 hours each. FastQ MinION files were subjected to quality control, trimming, and reference assembly using Minimap2 plugin implemented in Geneious Prime software v. 2021.2.2 (Biomatters Ltd., Auckland, New Zealand).

### 2.4. Sequence and Phylogenetic Analysis

Sequence analysis was performed using Geneious Prime software (v. 2021.2.2, Biomatters Ltd., Auckland, New Zealand). The sequences generated in this study were aligned and compared to each other and to those of hepaciviruses published in GenBank database, using the Multiple Alignment using Fast Fourier Transform (MAFFT) plugin implemented into the Geneious Prime software [23]. Phylogenetic analysis of 5′UTR, NS3, and NS5B genomic regions, as well as of the full-length polyprotein genomic region, were conducted with MEGA-X version 10.0.5 software [24] using the maximum likelihood method, the General Time Reversible model with a gamma distribution and invariant sites and bootstrapping up to 1000 replicates.

### 2.5. Nucleotide Sequence Accession Numbers

The complete EqHV genomic sequences of the strains ITA/2021/142m, ITA/2021/359 and ITA/2021/142o generated in this study were deposited in GenBank under the accession numbers ON653391–ON653393, respectively.

## 3. Results and Discussion

Starting from March 2021, we performed virological investigations in a small stable where clinical signs of liver disease were recorded. By RT-(q) PCR, three horses with clinical signs and clinical pathology changes suggestive of liver disease (#136, #141, and #142) tested positive to EqHV. In the following months, two horses (#183 and #536) acquired the infection after their introduction to the stable (Supplementary Figure 1). Furthermore, horse #359 tested positive one month after its entry in the stable, but sampling delay did not allow us to understand if the animal was infected just before its arrival in the stable. The virus was never detected in urine, feces, nasal, and oropharyngeal samples collected in parallel with the serum samples from the viremic horses. One of the horses testing positive to EqHV at their entry into the stable (horse #142) continued to be viremic throughout the whole observation period, suggesting a pattern of chronic infection.

The samples were screened for several equine pathogens listed earlier (panel HP) testing negative.

To date, the transmission routes of EqHV are not completely known. Experimental and iatrogenic transmission of EqHV by means of infected blood and blood products has been demonstrated as well as vertical transmission. However, the high prevalence of the virus, especially in some geographical regions [16–18], cannot be justified neither by parenteral transmission [14] nor by occasional vertical transmission [15]. Since many known flaviviruses are horizontally transmitted between hematophagous arthropods and vertebrate hosts, this transmission route has been investigated for EqHV. A large screening of over 5000 mosquitoes in areas with high EqHV prevalence failed to detect the virus [25], suggesting that mosquitoes are unlikely to play a role as vectors of this flavivirus. Recently, Shao et al. [26] identified the presence of bovine hepacivirus strains in ticks collected from cattle, in China. Likewise, in Australia a novel hepacivirus species was detected in ticks collected from bandicoots [27]. In both cases, the low abundance of the hepaciviral genome suggested that the ticks could serve as a mechanical carrier rather than a biological vector for these viruses. Therefore, further studies are needed to proper address the role of vectors in EqHV transmission.

Recent studies have highlighted the presence of viral RNA in nasopharyngeal swabs, but the collected data were not sufficient to prove airborne transmission of the virus, although this was not excluded [15]. In our study, six out of thirteen (46.2%) trotting horses tested positive to EqHV (46.2%). In particular, three EqHV-positive animals were already present in the stable at the beginning of our observational study, whilst two horses acquired the infection a few months after their introduction, suggesting a horizontal transmission of EqHV. Chronically infected animal (horse #142) was considered the possible source of infection for other animals.

**Figure 1:**
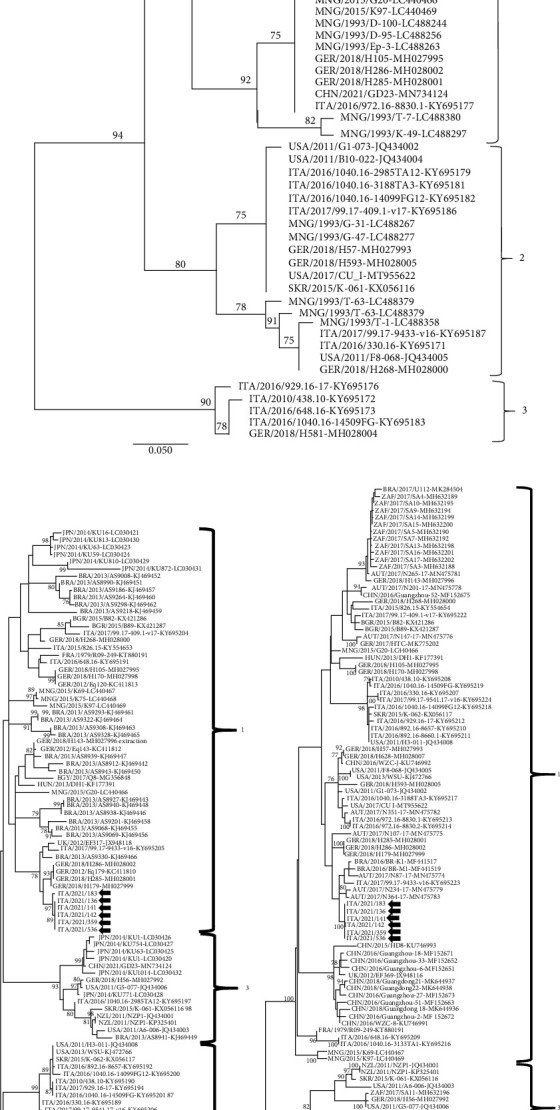
Phylogenetic trees based on three conserved genomic regions, (a) 5′UTR, (b) NS3 and (c) NS5B, of equine hepacivirus (EqHV) strains retrieved from the GenBank database and identified in this study. GenBank accession numbers are provided for reference strains. The trees were generated using the maximum likelihood method, general time reversible model with a gamma distribution and invariant sites and bootstrapping up to 1000 replicates. Bootstrap values ≥ 75% are shown. Italian EqHV strains generated in this study are indicated by black arrows. Scale bar indicates nt substitutions per site.

In order to assess this hypothesis, multitarget sequence and phylogenetic analysis (5′UTR, NS3, and NS5B genomic regions) of the generated sequences were performed. All EqHV strains circulating in the stable segregated within Subtype 1 (Figures 1(a)–1(c)) and were genetically highly related to each other, with more than 99% nucleotide (nt) sequence identity. Overall, the history of the stable and the temporal sequence of infections, together with the genetic conservation among the detected viruses, strongly suggest the contagious nature of the virus and horizontal transmission by either direct or indirect contact.

To date, the clinical importance of infection by EqHV, if any, is still unclear, although it appears to be minimal despite the wide distribution of the virus. In our study, all EqHV-positive trotting horses presented poor agonistic performance, even after they tested negative for EqHV. Four liver-specific parameters (AST, ALT, ALP, and GGT) were measured in order to evaluate the hepatopathy (Supplementary Table 1). At least two parameters, AST and GGT, consistently fell outside the reference ranges in all animals. This is not in agreement with previous studies [9, 10, 17] that reported liver markers within the reference ranges, with only few exceptions. Even if displaying increased levels of liver enzymes, all the EqHV-positive trotting horses did not show clinical signs of hepatitis. Unlike HCV infection in humans, at least 60% of the horses in the outbreak were able to clear EqHV infection. Thus, it could be speculated that this high rate of viral clearance among horses together with their average lifespan (25–30 years) may result in low levels of hepatic injury and lack of specific clinical signs [28].

It is well known that genomes of RNA viruses mutate at extremely high rates due to frequent error-prone replication cycles. Marked genetic diversity is a prominent feature of HCV strains. Interhost variability accounts for 8 genotypes and 105 subtypes detected in surveillance studies worldwide [29]. Intrahost variability accounts for a heterogeneous population of closely related variants (quasispecies) present in an infected patient [30]. HCV genotypes differ by over 30% at the nt level, whilst HCV subtypes by 20–25%. Intrahost quasispecies genetic diversity is estimated to be over 1–3% [31]. Thus far, molecular studies on EqHV strains have revealed a low genetic variation among virus strains [28], not comparable to what observed for HCV. This may be due to a rather short evolutionary history of EqHVs with a recent bottleneck event. The evolution of intrahost viral populations has not been studied in detail for EqHV yet.

In our study, we had the opportunity to analyze the complete viral sequences from a persistent infected horse (# 142), comparing the first (in March) and the last (in October) samplings. Analysis of the full-length genome sequence showed that ITA/2021/142m (GenBank accession ON653391) and ITA/2021/142o (GenBank accession ON653393) viruses were very similar (99.7% nt identity). There were only 26 nt changes resulting in 11 nonsynonymous nt substitutions (Table 1). The region producing the most diversities (coordinates: 434–561) encompassed the N-terminal half of the E2 protein. This is consistent to what observed for HCV which displays a hypervariable region at the N-terminus of E2 protein. The evolution of diversity in this region has been shown to correlate with the outcome of HCV infection [32] as well as the severity of hepatic injuries. Whether the substitutions we observed in EqHV are the result of a random drift or are influenced by host immune responses and may contribute to virus persistence is hard to predict in the absence of further data. Moreover, on full-length genome sequencing, strain ITA/2021/359 (GenBank accession ON653392) showed a 99.8% nt identity to strains ITA/2021/142m and ITA/2021/142o with which the strain clustered in the subtype 1 of EqHV (Supplementary Figure 2).

A major limit of this study was that we could not monitor and sample the horse with persistent infection for a longer period, thus missing the opportunity to track viral quasispecies diversification on a larger time span.

Finally, little is known about the potential risk factors and transmission routes of EqHV. The horizontal transmission, if confirmed, would explain and justify the observed prevalence data of the virus and will ensure the adoption of adequate measures of prophylaxis in horse stables, especially for high-risk categories, such as trotting horses. Also, the development of new diagnostic tools, such as serological tests, will help obtaining more accurate data on the prevalence of the virus as well as on the transmission patterns.

## Figures and Tables

**Table 1:** Table of variants in persisted infected horse #142 by comparative full-length genome sequence analysis between ITA/2021/142m and ITA/2021/142o strains. The aa were compared based on their R-group properties. “−” indicates no difference in the aa properties and “+” indicates that the aa belonged in different R-groups.

Genomic position (aa)	ORF	Nucleotide change^*∗*^	Amino acid change	aa properties
434	E2	**T**CC⟶**C**CC	Ser ⟶ Pro	+
437	E2	C**C**G⟶C**T**G	Pro ⟶ Lau	−
440	E2	A**A**A⟶A**G**A	Lys ⟶ Arg	−
444	E2	**G**AC⟶**A**AC	Asp ⟶ Asn	+
470	E2	A**G**G⟶A**A**G	Arg ⟶ Lys	−
477	E2	G**C**G⟶G**T**G	Ala ⟶ Val	−
561	E2	TG**C**⟶TG**G**	Cys ⟶ Trp	+
1309	NS3	G**GT**⟶G**TG**	Gly ⟶ Val	−
1310	NS3	**A**TT⟶**G**TT	Ile ⟶ Val	−
2235	NS5A	A**G**C⟶A**A**C	Ser ⟶ Asn	+
2261	NS5A	C**G**G⟶C**A**G	Arg ⟶ Gln	+

^*∗*^In bold the nucleotide substitutions. E2 envelope glycoprotein 2; NS3 nonstructural protein 3; NS5A nonstructural protein 5A.

## Data Availability

The nucleotide sequences that support the findings of this study will be openly available in the GenBank database at https://www.ncbi.nlm.nih.gov/genbank/ under Accession nos. ON653391, ON653392, and ON653393. The rest of the data that support the findings of this study are available from the corresponding author upon reasonable request.

## Supplementary Materials

Supplementary Materials Supplementary Table 1. Biochemical profiles of the horses tested positive for EqHV after their entrance in the stable. In bold values exceeding the normal upper limits are indicated. Supplementary Table 2. List of oligonucleotides used in RT-PCR protocols for sequencing of genomic fragments. Amplicon sizes are referred to the sequence of EqHV strain AK-2012 NPHV-NZP-1 (GenBank accession no. JQ434001). Supplementary Figure 1. Viral loads of horses (A–F) found positive for equine hepacivirus. A to C indicate the horses already present in the stable at the beginning of our investigation. D to F indicate the horses that were introduced subsequently in the stable, with the black arrows indicating the time of entrance. Black asterisks indicate the first sampling of the newly introduced animals. Supplementary Figure 2. Phylogenetic tree based on the full-length polyprotein genomic region of equine hepacivirus (EqHV) strains retrieved from the GenBank database and identified in this study. GenBank accession numbers are provided for reference strains. The trees were generated using the maximum likelihood method, General Time Reversible model with a gamma distribution and invariant sites and bootstrapping up to 1000 replicates. Bootstrap values ≥ 75% are shown. Italian EqHV strains ITA/2021/142m (GenBank accession no. ON653391), ITA/2021/142o (GenBank accession no. ON653393) and ITA/2021/359 (GenBank accession no. ON653392) generated in this study are indicated by black arrows. Scale bar indicates nt substitutions per site.
