# Supplementary material for: Description of an Equine Hepacivirus Cluster in a Horse Stable in Italy
Source: Transbound Emerg Dis. 2023 May 5;2023:5251034. doi: 10.1155/2023/5251034 (PMC12017193; doi:10.1155/2023/5251034)

**Supplementary material**

**Supplementary Tables**

**Supplementary Table 1.** Biochemical profiles of the horses tested positive for EqHV after their entrance in the stable. In bold values exceeding the normal upper limits are indicated.

| Animal | Gender | Age | Date of entry | ALT  (4-16 UI/L) | AST  (170-330 UI/L) | ALP  (160-380 UI/L) | GGT  (8-25 UI/L) |
| --- | --- | --- | --- | --- | --- | --- | --- |
| 136 | M | 3 years | 01/03/2021 | **20** | **1120** | 228 | 21.6 |
| 141 | M | 18 months | 03/03/2021 | 16 | **582** | **596** | **57.6** |
| 142* | M | 3 years | 03/03/2021 | **111** | **2679** | 300 | **46.8** |
| 183 | M | 6 years | 25/03/2021 | **19** | **666** | 356 | **43.4** |
| 359 | M | 3 years | 10/05/2021 | 13 | **396** | 280 | **30.2** |
| 536 | F | 7 years | 08/09/2021 | **25** | **1002** | **448** | **60.6** |

* Horse with persistent infection profile

*ALT* alanine transaminase; *AST* aspartate transaminase; *ALP* alkaline phosphatase; *GGT* gamma glutamyl transpeptidase;

**Supplementary Table 2.** List of oligonucleotides used in RT-PCR protocols for sequencing of genomic fragments. Amplicon sizes are referred to the sequence of EqHV strain AK-2012 NPHV-NZP-1 (GenBank accession no. JQ434001).

| **Gene target** | **Primer** | **Sequence 5’ - 3’** | **Amplicon size** | **Reference** |
| --- | --- | --- | --- | --- |
| 5’ UTR - NS3 region | Quanti5UF1 | GAGGGAGCTGRAATTCGTGAA | 4388 bp | (Burbelo et al., 2012) |
|  | EQNS3OAS | TAGTAGGTBACAGCRTTAGCYCC |  | (Lyons et al., 2012) |
|  | 201F | AGCTGRAATTCGTGAATYYGTGA | 4383 bp | (Elia et al., 2017) |
| NS3 - NS5B region | EQNS3OS | ATWTGTGATGARTGCCAYAGYAC | 4386 bp | (Lyons et al., 2012) |
|  | EQNS5BOIS | ACTRTGACTRATYGTYTCCCAACTCG |  |  |
|  | EQNS3IS | TCYAARGGTGTDAAGCTTGTTGT | 4107 bp |  |
|  | EQNS5BIAS2 | TCRTCTTCCTCRACGCCYTTRCTGG |  |  |

**Supplementary Figures**

**Supplementary Figure 1.** Viral loads of horses (A-F) found positive for equine hepacivirus. A to C indicate the horses already present in the stable at the beginning of our investigation. D to F indicate the horses that were introduced subsequently in the stable, with the black arrows indicating the time of entrance. Black asterisks indicate the first sampling of the newly introduced animals.


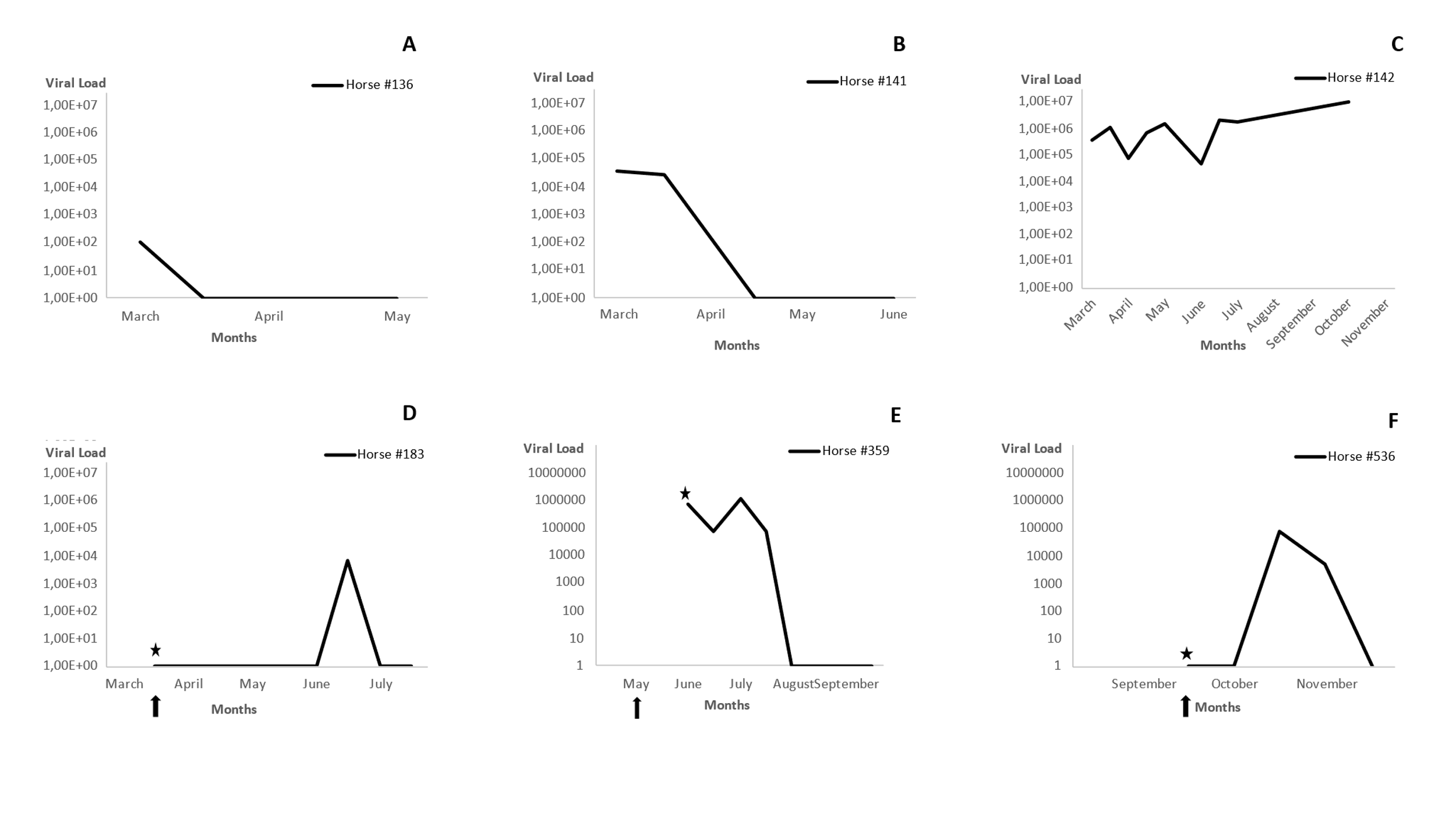


**Supplementary Figure 2.** Phylogenetic tree based on the full-length polyprotein genomic region of equine hepacivirus (EqHV) strains retrieved from the GenBank database and identified in this study. GenBank accession numbers are provided for reference strains. The trees were generated using the maximum likelihood method, General Time Reversible model with a gamma distribution and invariant sites and bootstrapping up to 1000 replicates. Bootstrap values ≥ 75% are shown. Italian EqHV strains ITA/2021/142m (GenBank accession no. ON653391), ITA/2021/142o (GenBank accession no. ON653393) and ITA/2021/359 (GenBank accession no. ON653392) generated in this study are indicated by black arrows. Scale bar indicates nt substitutions per site.


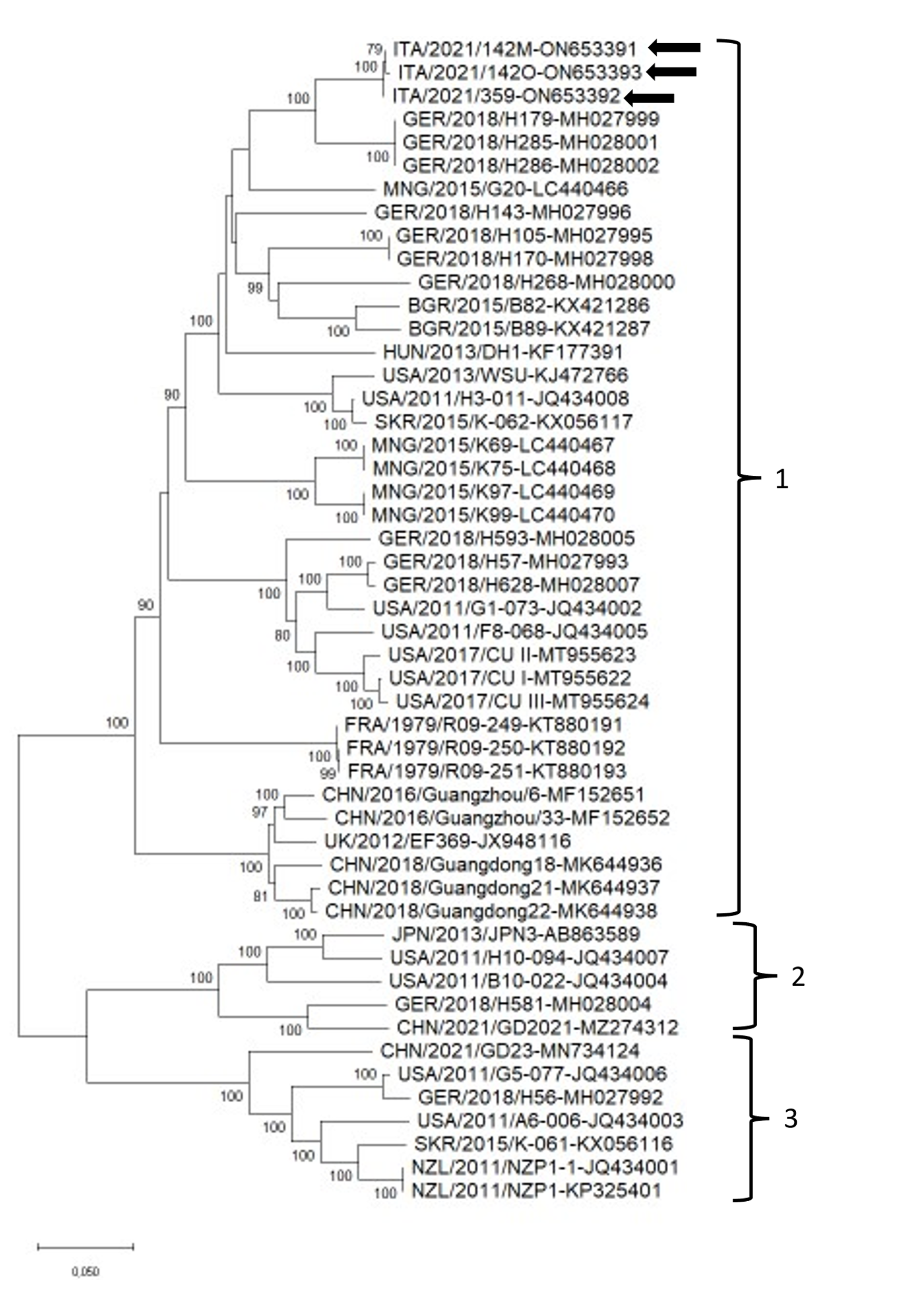

Supplement: Supplementary Materials — Supplementary Table 1. Biochemical profiles of the horses tested positive for EqHV after their entrance in the stable. In bold values exceeding the normal upper limits are indicated. Supplementary Table 2. List of oligonucleotides used in RT-PCR protocols for sequencing of genomic fragments. Amplicon sizes are referred to the sequence of EqHV strain AK-2012 NPHV-NZP-1 (GenBank accession no. JQ434001). Supplementary Figure 1. Viral loads of horses (A–F) found positive for equine hepacivirus. A to C indicate the horses already present in the stable at the beginning of our investigation. D to F indicate the horses that were introduced subsequently in the stable, with the black arrows indicating the time of entrance. Black asterisks indicate the first sampling of the newly introduced animals. Supplementary Figure 2. Phylogenetic tree based on the full-length polyprotein genomic region of equine hepacivirus (EqHV) strains retrieved from the GenBank database and identified in this study. GenBank accession numbers are provided for reference strains. The trees were generated using the maximum likelihood method, General Time Reversible model with a gamma distribution and invariant sites and bootstrapping up to 1000 replicates. Bootstrap values ≥ 75% are shown. Italian EqHV strains ITA/2021/142m (GenBank accession no. ON653391), ITA/2021/142o (GenBank accession no. ON653393) and ITA/2021/359 (GenBank accession no. ON653392) generated in this study are indicated by black arrows. Scale bar indicates nt substitutions per site. [file 5251034.f1.docx]
